# Keeping eyes peeled: guppies exposed to chemical alarm cue are more responsive to ambiguous visual cues

**DOI:** 10.1007/s00265-016-2076-4

**Published:** 2016-02-23

**Authors:** Jessica F. Stephenson

**Affiliations:** School of Biosciences, Cardiff University, Cardiff, CF10 3AX UK; Center for Adaptation to a Changing Environment (ACE), ETH Zürich, Institute of Integrative Biology, 8092 Zürich, Switzerland; Department of Aquatic Ecology, EAWAG, Swiss Federal Institute of Aquatic Science and Technology, 8600 Dübendorf, Switzerland

**Keywords:** Alarm cue, Cross-modal sensory interaction, Multisensory cues, *Poecilia reticulata*, Threat-sensitive behavior

## Abstract

**Abstract:**

Information received from the visual and chemical senses is qualitatively different. For prey species in aquatic environments, visual cues are spatially and temporally reliable but risky as the prey and predator must often be in close proximity. Chemical cues, by contrast, can be distorted by currents or linger and thus provide less reliable spatial and temporal information, but can be detected from a safe distance. Chemical cues are therefore often the first detected and may provide a context in which prey respond to subsequent ambiguous cues (“context hypothesis”). Depending on this context, early chemical cues may also alert prey to attend to imminent cues in other sensory modalities (“alerting hypothesis”). In the context of predation risk, for example, it is intuitive that individuals become more responsive to subsequent ambiguous cues across sensory modalities. Consistent with the context hypothesis, guppies, *Poecilia reticulata*, exposed to conspecific alarm cue reduced activity, a classic fright response among fish, in response to a water disturbance more than those exposed to cues of unharmed conspecifics or a water control. Despite this reduction in activity, guppies exposed to alarm cue were more attentive to visual cues than those exposed to the other chemical cues, as predicted by the alerting hypothesis. These responses contrasted with those of guppies exposed to chemical cues of undisturbed, unharmed conspecifics, which were relatively unaffected by the disturbance. This is the first study indicating that unambiguous cues detected by one sensory modality affect animal responses to subsequent ambiguous multimodal cues.

**Significance statement:**

In moving water, chemical cues can be detected over longer distances than visual cues; they may therefore be detected first and alert animals to imminent visual cues. This effect is likely to be particularly important if these chemical cues are indicative of predation. I investigated how different chemical cues affect (1) guppy response to an ambiguous water disturbance and (2) their responsiveness to subsequent ambiguous visual cues. Guppies based their responses to ambiguous cues on the context implied by chemical cues: those exposed to chemical cues indicative of predation reduced activity, a classic fright response, but increased responsiveness to visual cues, relative to those exposed to control chemical cues. This is the first study to show that unambiguous cues detected by one sense affect animal responses to ambiguous cues detected by other senses.

**Electronic supplementary material:**

The online version of this article (doi:10.1007/s00265-016-2076-4) contains supplementary material, which is available to authorized users.

## Introduction

Information received from the visual and chemical senses is qualitatively different. For prey species in aquatic environments, visual cues of predation are spatially and temporally reliable but risky as the prey and predator often have to be in close proximity due to short visualization distances (Lythgoe 1979). Chemical cues, by contrast, can be distorted by currents or linger and thus provide less reliable spatial and temporal information, but can be detected from a safe distance or while in hiding (Brown and Magnavacca 2003). For this reason, chemical cues can be considered long-distance cues in moving water (Dusenberry 1992) and may often be the first cue an animal receives (McLennan 2003). Previous studies indicate that the first cue received alerts the recipient to the potential presence of a second cue, enhancing the detectability and discriminability of the second cue and therefore reducing the chance of overlooking vital information (Rowe 1999, Rowe and Guildford 1999). This effect may be particularly strong when the cues are detected by different sensory modalities (“multimodal”; Rowe 1999).

The detection of predation risk is an ideal process with which to test hypotheses relating to multimodal sensory ecology: prey response to cues of predation risk is vital for survival, but responding to non-threatening cues in the same way is a waste of resources (Helfman 1989, Lima and Dill 1990), so prey are likely to be under strong selection to use all available information to make appropriate decisions (Munoz and Blumstein 2012). As a result, the interaction between sensory modalities, particularly the visual and chemosensory systems, in prey response to the cues of predation risk has received considerable research attention (reviewed by Munoz and Blumstein 2012). Many studies of this process in aquatic systems have used the chemical cue I use in this study, “alarm cue,” which is released from fish skin damaged during predation events and, if detected, provides reliable information about predation risk in the immediate environment regardless of predator identity (Brown 2003). Typically, fish exposed to alarm cue reduce activity and increase shoaling behavior (Brown 2003, Ferrari et al. 2005, Whitlock 2006). In many fishes, the innate response to alarm cue is sufficiently strong that a single associative conditioning event can enhance existing antipredator behavior (e.g., Berejikian et al. 1999) and condition a response to the odors of novel predators (Ferrari et al. 2005), non-predatory fish (Larson and McCormick 2005), sound (Wisenden et al. 2008), areas of habitat in the wild (Kim et al. 2011), and non-biological visual cues (Hall and Suboski 1995, Yunker et al. 1999). Additionally, the concentration of chemical alarm cue can indicate the level of predation risk on a temporal or spatial scale; several species use cue concentration to change their behavior in a “threat-sensitive” way, i.e., as the level of perceived risk increases, inferred from the concentration of alarm cue, the intensity of response also proportionally increases (Helfman 1989; e.g., ambon damselfish, *Pomacentrus amboinensis*, see Lönnstedt and McCormick 2011; Trinidadian guppies, *Poecilia reticulata*, see Brown et al. 2009; and Atlantic salmon, *Salmo salar*, see Hawkins et al. 2007).

Studies using chemical alarm cue have led to the development of several models of multimodal cue used in predation risk assessment. Hartman and Abrahams (2000) proposed the sensory compensation model, that one sensory modality should take precedence over others as the primary source of risk assessment information. This model explains their observation and that of others that fish in conditions preventing the use of visual cues (hypothesized as the more important cues) typically show stronger responses to chemical alarm cue and predator cues (Hartman and Abrahams 2000, Leduc et al. 2010, Leahy et al. 2011). More recently, however, studies have revealed the opposite pattern: fish unable to use chemical alarm cue show stronger responses to visual cues (e.g., Elvidge et al. 2012).

Further empirical work has confirmed that visual and chemical cues instead act synergistically to determine fish response to predation risk and provide qualitatively and quantitatively different information: the sensory complement model (Ferrari et al. 2008). Specifically, visual and chemical cues combined provide prey with more information about local risks than either visual or chemical cues in isolation. For example, the response of glowlight tetras, *Hemigrammus erythrozonus*, to visual cues of a predator is greater when fish are pre-exposed to alarm cue than the response to either the visual or chemical cue in isolation (Wisenden et al. 2004), even when pre-exposure is to concentrations of alarm cue too low to elicit an overt behavioral responses (Brown et al. 2004). Although this body of work demonstrates that cues from different sensory modalities interact in determining fish behavior, in each case, the visual and chemical cues were unambiguously those of predation or a predator (e.g., chemical alarm cue paired with a model predator; reviewed by Munoz and Blumstein 2012). No study, to my knowledge, has yet investigated how threatening cues detected by one modality affect an individual’s response to subsequent ambiguous cues in other modalities.

Here, I use the guppy, *P. reticulata*, to test two outstanding hypotheses in this field. Guppies are typically considered a highly visual species and have excellent vision (Anstis et al. 1998), but recent research has revealed that they also use chemical cues: they show graded responses proportional to the concentration of alarm cue presented (Brown et al. 2009); respond most strongly to alarm cue from individuals from their own population (Brown et al. 2010); and can use chemical cues to assess the sex (Shohet and Watt 2004), reproductive status (Brask et al. 2012), and health (JFS, unpublished data) of conspecifics. In this experiment, I exposed guppies to either one of two concentrations of conspecific chemical alarm cue (100 or 10 %), the chemical cues of unharmed conspecifics (as a non-threatening control cue), or a water control. Given that chemical cues are often the first received by aquatic organisms (McLennan 2003), I tested whether guppies use these cues to infer the context (sensu Hebets and Papaj 2005) in which to respond to subsequent, ambiguous cues in different modalities: a water disturbance and visual cue (the “context hypothesis”). These cues can be considered ambiguous because in natural guppy habitat of shallow, gravel-bottomed streams, water disturbance and visual cues could come from a number of different sources: for example the movement of conspecifics, predators, or abiotic processes. Second, I test whether, particularly in the “predation risk” context, exposure to chemical cues alerts (sensu Hebets and Papaj 2005) guppies to visual cues using a standard test of visual sensitivity (“alerting hypothesis”): the “optomotor” response. The optomotor response can be elicited using the movement of alternating black and white stripes; fish swim to remain in the same place relative to the stripes. This response has been used to measure the visual sensitivity of a number of fish species, including guppies (Anstis et al. 1998). In a natural setting, this response enables fish to maintain their position relative to the substrate in flowing water, or to maintain their position in a group.

Consistent with the context hypothesis, I predicted that guppies exposed to chemical alarm cue would show a reduction in activity following the water disturbance, proportional to cue concentration, whereas those exposed to other chemical cues would not. Following the alerting hypothesis, I predicted that alarm cue-exposed guppies would be more responsive to visual cues (i.e., spend a higher proportion of time following the stripes) than those exposed to other chemical cues. The results broadly supported these predictions.

## Methods

### Fish origin and maintenance

Test fish used in this study were wild caught in the Caura River, Trinidad, in June 2012 (UTM 20 P; E: 679527.7 m, N: 1180376.4 m, based on WGS84 Datum; elevation 112 m). They were shipped to Cardiff University (Cefas APB authorization number CW054-D-187A), treated for infection using Binox® (nitrofurazone; Jungle Laboratories Corporation®, Cibolo, Texas), and held for 3 weeks before testing. Fish were housed in 70-L aquaria at 24 ± 1 °C, on a 12-h light/12-h dark lighting schedule (overhead fluorescent lighting) and fed daily on Aquarian® flakes supplemented with *Artemia* and bloodworm. Each tank had pea gravel substrate, an under gravel filter and standardized enrichment.

### Chemical cue production

All chemical cues were produced in two batches. In each batch of alarm cue and the cue of unharmed conspecifics (“fish cue”), mature laboratory-bred females from the same wild population as the test fish were selected as donors. Exclusively, female donors were used because males of some fish species produce alarm cue intermittently (e.g., Smith 1973). While this is an interesting phenomenon, it was not the focus of this study, so only donors known to reliably produce alarm cue, i.e., females (Brown et al. 2009), were selected. These donors were held separately from the test fish at all times and had been in the laboratory for several years before the test fish arrived; there was therefore no possibility of familiarity between the test and donor fish. To make fish cue, seven donors per batch were held together for 20 h in 2 L blank dechlorinated water. They were not fed during this isolation to avoid the cues becoming contaminated with uneaten food or feces and were subsequently returned to breeding tanks and were not used as either alarm cue donors or test fish. The holding water was divided into 10-mL aliquots and frozen until required. Alarm cue production followed the protocol of Brown et al. (2009). Seven donors were cold anaesthetized and immediately decapitated. The tail and viscera were also removed, leaving skeletal muscle and skin. All carcasses were added to 50 mL of chilled, dechlorinated water, homogenized and the solution filtered through glass wool. The concentration was adjusted to 0.1 cm^2^ of skin/mL, following Brown et al. (2009). This 100 % alarm cue solution was either divided into 10-mL aliquots and frozen at −20 °C until required, or diluted with dechlorinated water to make a 10 % alarm cue solution which was then similarly divided and frozen. The same volume of “control” dechlorinated water was held overnight, divided, and frozen until use.

### Optomotor apparatus

**Fig. 1:**
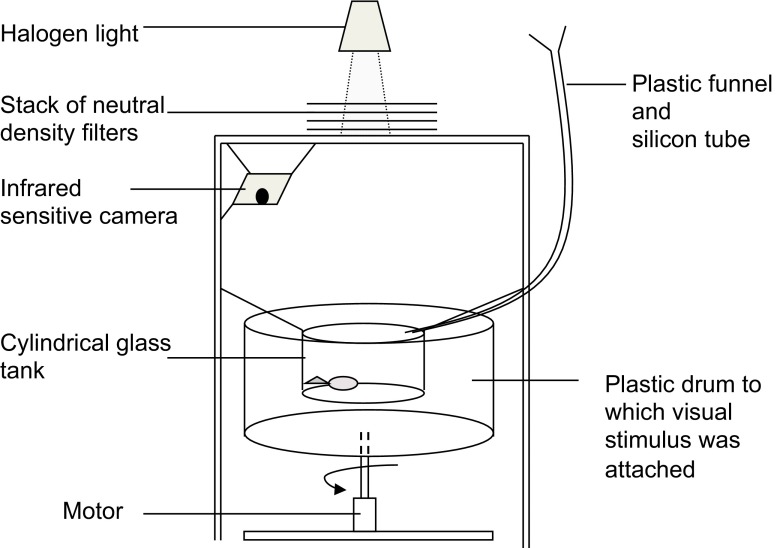
The apparatus used to elicit the optomotor response of guppies and to use this response to test how visual behavior was affected by chemical cues

The optomotor apparatus was adapted from Stephenson et al. (2011, 2012) and consisted of a cylindrical glass tank (diameter 18 cm, depth 10 cm) suspended from a steel frame (Fig. 1) The tank was surrounded by a drum (diameter 28 cm, depth 14 cm), which could be rotated in either direction by a motor at a constant speed of 10 rpm. The drum supported a visual cue consisting of alternating black and white stripes, each covering 20° of the arc of the drum circumference. The fish were viewed and behavior recorded using an infrared-sensitive video camera (Henelec 300c CCTV IR) supported from the top of the frame. The sides of the frame were covered in blackout fabric, and the top was covered with an MDF board. A 1-cm-diameter circular hole was drilled into the board (“light hole”), and a halogen fiber optic light source (Schott KL 1500 LCD) was positioned above it to provide 1.5 lx of light at the surface of the water (approximately 2 × 10^17^ photons/s/m^2^ using the calculations described by Stephenson et al. 2011, 2012). In order to attenuate the light further, 7 × 7 cm squares of neutral-density (ND) filters (LEE filters; one layer of 299 and six of 209; nominal absorbances 1.2 and 0.3, respectively) were laid over the light hole. This light level was chosen during preliminary work as one at which the fish could see and respond to visual cues, but their response was limited compared to that at ambient light levels. This light level therefore provided the opportunity to observe any increase in visual sensitivity above the baseline level due to olfactory stimulation, as has been observed in zebrafish (Stephenson et al. 2011, 2012). During trials, chemical cues were introduced to the experimental tank in water using separate funnels and Nalgene® tubing. The tube was fed through a covered hole in the screen surrounding the frame, and the end hung 2 cm above the surface of the water in the experimental tank. The experimental room was held at 24 ± 0.5 °C.

### Experimental protocol

Fish to be tested were held individually overnight in opaque white 1-L tanks and were not fed during their isolation. These tanks were wiped with 70 % ethanol and rinsed thoroughly with dechlorinated water between uses. The experimental tank was filled with dechlorinated water to a depth of 4 cm, and a naïve test fish was added. An opaque board was placed over the light hole for 25 min to allow the fish to acclimatize and dark-adapt. Each trial began with the drum being rotated at 10 rpm for 30 s in each direction for 2 min. During the third minute, the chemical cue (100 % alarm cue, 10 % alarm cue, fish cue, or control) was injected into the tank. Because the input tube hung 2 cm above the surface of the water, chemical cue input caused a disturbance at the surface of the water and therefore visual and mechanosensory as well as chemical cues. The rotation of the drum (30 s in each direction) was repeated during the four minutes immediately following chemical cue input. The visual cues were therefore rotated following this pattern during minutes 1 and 2 of each trial and in minutes 4 to 7 (i.e., the four minutes following chemical cue input), but not during minute 3 (in which chemical cue input took place). At the end of each trial, the chemical cue input tube was rinsed with dechlorinated water. The fish was removed, weighed and measured, and returned to a breeding tank. Equal numbers of male and female guppies were tested using each of the four chemical cues. Ten replicates of these eight treatments were completed over the course of 14 days. Trials were run such that no treatment was repeated before a complete set of the eight treatments, or “experimental block,” had been run. Treatment order was randomized within block, and the order of the treatments was changed between blocks following a Latin square design.

### Data analysis

**Fig. 2:**
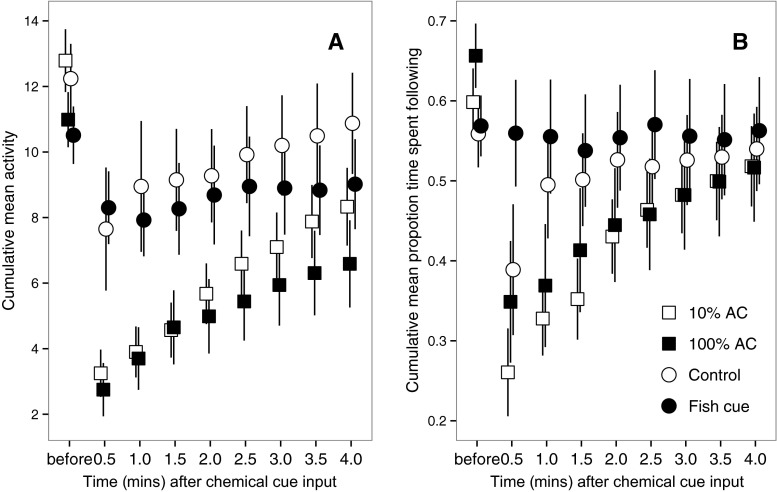
Guppy activity level depended on the nature of the chemical cue, and the time since chemical cue input (**a**), whereas the proportion of time fish spent following the visual cues depended on time since chemical cue input alone (**b** alarm cue (*AC*) of different concentrations; dechlorinated water (*control*), or the cues of unharmed conspecifics (*fish cue*)). *Before* refers to the mean value across all four time points before the chemical cue input. The analyses described in the main text were conducted on the transformed raw data, but raw data were converted to cumulative values for these plots for clarity. *Error bars* are the standard errors of the means

The proportion of each 30-s period that the fish spent following the stripes was calculated from the trial videos using JWatcher™ 1.0 (www.jwatcher.ucla.edu/). The observer additionally scored the number of times the fish swam through a quarter of the tank (e.g., a full circuit of the tank would count as an activity score of 4, as would half the tank in one direction plus half in the other) as a measure of activity. To minimize observer bias, blinded methods were used during the video analysis. For the four minutes immediately after the input of the chemical cues, both fish activity (square-root transformed; model 1 in Table 1) and the proportion of each minute the fish spent following the stripes (arcsine transformed; model 2 in Table 1) were used as the response variables in two linear mixed models in the lme4 package in R 3.0.2 (LMM; Gaussian error family with identity link function; Bates et al. 2013, R Core Team 2013). In each model, fish identity was included as the random term to account for repeated measures through time. The experimental block in which a trial was conducted was additionally included as a random term. The sex of the fish, the chemical cue to which it was exposed (“treatment”), activity (model 2 only), standard length, time since the chemical cue had been input, and the experimental block in which the trial was conducted were all included as fixed effects, as well as the two-way interactions between them (Table 1). Non-significant fixed effects were sequentially deleted from the starting models to minimize the Akaike’s information criteria (AIC), and only significant effects are reported. The supplementary material provides further details of these analyses, including histograms of the residuals of the full and final models and model tables at each step of simplification. These analyses were conducted on the raw (transformed) data, but the data were converted to cumulative values for Fig. 2 for clarity.

**Table 1:** Starting models used to test the hypothesis that chemical cues affect the way guppies respond to ambiguous disturbance and visual cues

Model and response variable	Error family	Link function	Main effects	Two-way interactions	Random effects
1. Activity (square-root transformed)	Gaussian	Identity	Length (n)	Time × length	Fish identity Block
			Sex (c)	Time × treatment^a^	
			Time (n)^a^	Time × sex	
			Treatment (c)^a^	Sex × length	
				Treatment × sex	
2. Proportion of time spent following the stripes (arcsine transformed)			Activity (n)^a^	Activity × time^a^	
			Length (n)	Activity × treatment^a^	
			Sex (c)^a^	Activity × sex	
			Time (n)^a^	Time × length	
			Treatment (c)^a^	Time × treatment	
				Time × sex^a^	
				Sex × length	
				Treatment × sex	

These starting models were simplified using backwards stepwise deletion of non-significant fixed effects to minimize the Akaike’s information criteria (AIC). Fixed effects were included as categorical (*c*) or numeric (*n*) variables

*Block* the experimental block in which a particular trial was conducted, *Treatment* the chemical cue to which the fish was exposed (100 % or 10 % alarm cue, fish cue, or control water), *Time* the experimental time elapsed since the introduction of the chemical cue

^a^Factors that remained in the final model (see also Tables 2 and 3)

## Results

Consistent with the context hypothesis, the chemical cue a fish was exposed to affected how its activity level changed through the four minutes following the disturbance caused by the chemical cue input (Fig. 2a; model 1 in Table 1: treatment × time interaction in Table 2: *F*_3, 483.2_ = 11.73, *P* < 0.0001). Post hoc tests showed that guppies exposed to the chemical cues of unharmed conspecifics and control water did not differ in activity level (*F*_1, 29.1_ = 0.67, *P >* 0.4), but showed significantly less of a decrease in activity level and recovered more quickly (*F*_1, 558_ = 24.31, *P* < 0.0001) than the two alarm cue treatment groups (which did not differ from one another: *F*_1, 29_ = 1.60, *P >* 0.2; Fig. 2a).

**Table 2:** The final model explaining variation in fish activity level (square-root transformed)

Parameter	Parameter level	Estimate	*F*	Degrees of freedom	*P* value
Intercept		1.90			
Time		0.12	120.88	1, 483.2	**<0.0001**
Treatment (reference control)	10 % alarm cue	−1.60	0.97	3, 67.16	0.412
	100 % alarm cue	−0.85			
	Unharmed conspecific	0.57			
Time × treatment (reference control)	Time × 10 % alarm cue	0.12	11.73	3, 483.2	**<0.0001**
	Time × 100 % alarm cue	0.03			
	Time × unharmed conspecific	−0.08			

Significant terms (at *α* = 0.05) in this final model are highlighted in bold

**Fig. 3:**
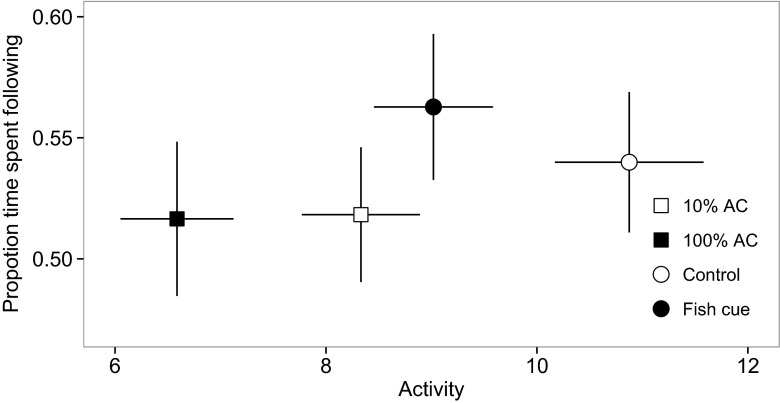
Guppies exposed to concentrated alarm cue (*100 % AC*) showed a significant reduction in activity level, but no significant decrease in the proportion of time they spent following the visual cues, relative to those exposed to dilute alarm cue (*10 % AC*), the cues of unharmed conspecifics (*fish cue*), or dechlorinated water (*control*). Data points show the raw data means across the four minutes following chemical cue input, and the *error bars* are the 95 % confidence intervals

**Fig. 4:**
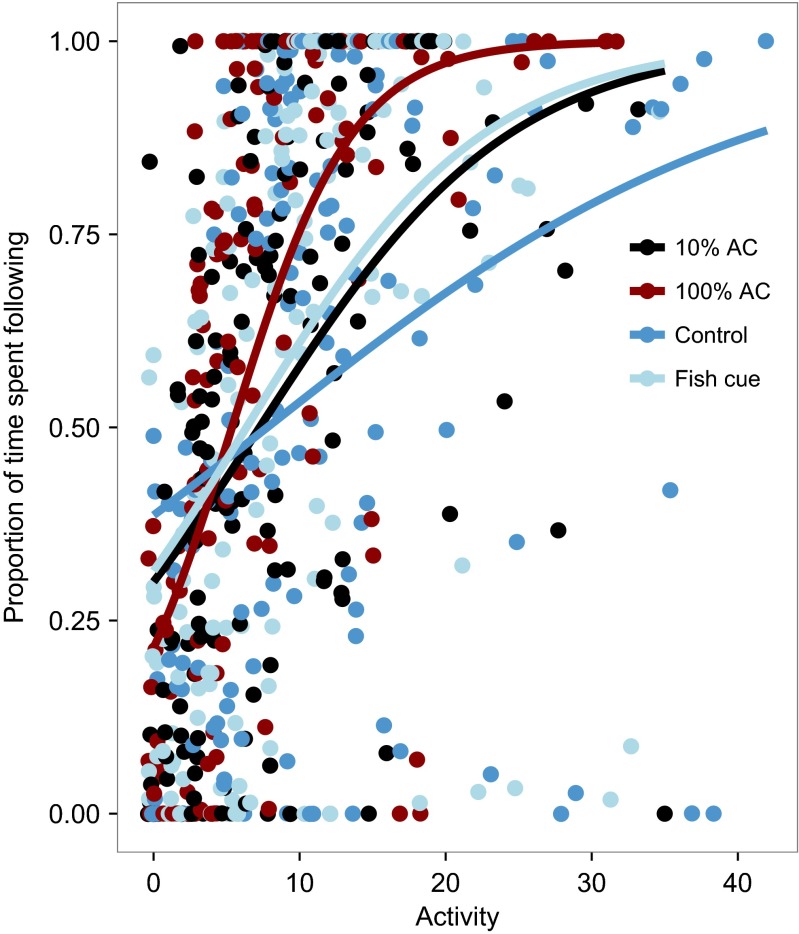
For a given activity level, guppies exposed to 100 % alarm cue spent significantly more time following the visual cues than those exposed to 10 % alarm cue, unharmed conspecifics, and dechlorinated water. There was no difference between the proportion of time guppies exposed to cues of unharmed conspecifics and 10 % alarm cue spent following the visual cues, but both groups spent more time following than those exposed to dechlorinated water (see main text for statistical tests). The *points* are the raw data and the *lines* are binomial regressions fitted to each treatment group

Exposure to 100 % alarm cue thus reduced fish activity levels, but it increased the extent to which this activity was focused on responding to the visual cues: there was no significant difference in the proportion of time fish exposed to different chemical cues spent following the visual cues over the whole four minutes after chemical cue input (Figs. 2b and 3; model 2 in Table 1: treatment main effect in Table 3: *F*_3, 64.6_ = 0.89; *P >* 0.4). Additionally, the positive correlation between activity level and response to visual cues was steepest among fish exposed to alarm cue (Fig. 4; model 2 in Table 1: activity × treatment interaction in Table 3: *F*_3, 535.2_ = 3.93, *P* = 0.009). These results support the prediction from the alerting hypothesis that fish exposed to alarm cue are more responsive to visual cues than those exposed to either the cues of unharmed conspecifics or dechlorinated water. Additionally, the proportion of time fish spent following the visual cues increased through time after the input of the chemical cue, but not among fish that remained highly active throughout (Fig. 2; model 2 in Table 1: activity × time interaction in Table 3: *F*_1, 563.4_ = 27.6, *P* < 0.0001).

**Table 3:** The final model explaining variation in the proportion of time fish spent following the stripes (arcsine transformed)

Parameter	Parameter level	Estimate	*F*	Degrees of freedom	*P* value
Intercept		−0.26			
Sex (reference female)		−0.10	1.66	1, 68.0	0.202
Time		0.06	7.97	1, 581.7	**0.005**
Activity		0.07	91.05	1, 544.1	**<0.0001**
Treatment	10 % alarm cue	−0.07	0.89	3, 64.6	0.45
	100 % alarm cue	−0.06			
	Unharmed conspecific	−0.06			
Fish weight		0.001	3.62	1, 43.3	0.063
Activity × treatment (reference control)	Activity × 10 % alarm cue	0.01	3.93	3, 535.2	**0.009**
	Activity × 100 % alarm cue	0.03			
	Activity × unharmed conspecific	0.01			
Activity × time		−0.005	27.6	1, 563.4	**<0.0001**

Significant terms (at *α* = 0.05) in this final model are highlighted in bold

## Discussion

As predicted by the context hypothesis, guppies used chemical cues to respond to a disturbance in a threat-sensitive manner. Those exposed to either concentration of conspecific alarm cue (10 or 100 %) reduced their activity level significantly more than those exposed to the chemical cues of unharmed conspecifics or dechlorinated water (Fig. 2a). Despite this difference in activity level, there was no overall difference between the groups exposed to the different chemical cues in their response to visual cues (Fig. 3), and the positive correlation between activity level and response to visual cues was steepest in fish exposed to alarm cue (Fig. 4). Consistent with the alerting hypothesis, these results indicate that guppies exposed to chemical alarm cue are more responsive to visual cues than those exposed to control chemical cues. Exposure to dilute and concentrated conspecific alarm cue increased visual responsiveness to the same extent, but the activity level of guppies exposed to the dilute cue recovered more quickly (Fig. 2a) and was higher overall (Fig. 3) than that of those exposed to the concentrated cue. Previous studies indicate that at concentrations below the “minimum behavioral response threshold” (Mirza and Chivers 2003), overt antipredator behaviors are not elicited and prey instead exhibit covert responses, such as changes in foraging posture (Foam et al. 2005), or the acquisition of novel predator cues (Ferrari et al. 2005). The results of the present study suggest that increased visual responsiveness could be a further covert response.

Guppies exposed to the chemical cues of unharmed conspecifics, in contrast to those exposed to chemical alarm cue, were not affected by the disturbance; both their activity level and response to the visual cues barely changed after the disturbance of the chemical cue input (Fig. 2). The guppy is a social animal; assessing risk through attending to the cues emitted by individuals in close proximity is a common feature of sociality across taxa, including fish (reviewed by Griffin 2004). The chemical cues of conspecifics can affect the extent to which fish respond to cues of predation risk. For example, rainbow trout, *Oncorhynchus mykiss*, in receipt of the chemical cues from undisturbed conspecifics show a reduced response to alarm cue compared to those exposed to cues from disturbed conspecifics (Ferrari et al. 2008). Further, fathead minnows, *Pimephales promelas*, trust the response of conspecifics to ambiguous cues more than their own learned response (Crane and Ferrari 2015). The results of the present study reflect that this process may act across sensory modalities: guppies in receipt of the chemical cues of unharmed, undisturbed conspecifics use this information to infer the non-threatening nature of the ambiguous water disturbance and subsequent visual cues and hence show no change in behavior.

Guppies therefore appear to use information from chemical cues to reduce the uncertainty inherent in ambiguous (and therefore unreliable) cues in other modalities. This process has recently been called “cue linking”: responding to unreliable cues only if they are linked to other cues that can increase their reliability (Ben-Ari and Inbar 2014). Recent research indicates cue linking may be widespread among animals; for example, humans infer the context of ambiguous video clips using the music that accompanies them (Blumstein et al. 2012). Similarly, aphids use hot, humid air as a reliable cue of the presence of a mammalian predator and respond to plant vibration either as further evidence of predator presence, or as wind action, based on the time lag between these two cues (Ben-Ari and Inbar 2014).

The present study additionally indicates that there may be differences in sensory physiology between fish families. Evidence from both electrophysiological and ethological studies indicate that the fish visual system is affected by chemical cues (Maaswinkel and Li 2003; Stephenson et al. 2011), including alarm cue (Stephenson et al. 2012). Whereas these studies demonstrated that visual sensitivity increases with chemical stimulation using the zebrafish and invoked the terminal nerve as the physiological pathway, I found no evidence of such an effect in guppies: following behavior never exceeded the pre-chemical cue level. Currently, all studies of the role of the terminal nerve in this interaction between sensory systems have been conducted on the cyprinids zebrafish, *Danio rerio* (see Maaswinkel and Li 2003; Stephenson et al. 2011, 2012) and goldfish, *Carassius auratus* (see Stell et al. 1984; Fujita et al. 1991); this study could therefore indicate that the results from these previous studies are not applicable to other families of fish.

My results indicate that guppy response to the visual cues increased as these cues were repeated through time; this pattern might be considered indicative of the fish becoming sensitized to the cues. “Behavioral sensitization” refers to increased responsiveness following arousal by rewarding or punishing experiences, with responsiveness increasing through repeated exposure, and is common to animals across taxa (e.g., honeybees, *Apis mellifera*, see Mallon et al. 2003; mice, *Mus musculus*, see Banasikowski et al. 2015; and humans, *Homo sapiens*, see Strakowski et al. 1996). However, I consider this explanation of the results unlikely: if sensitization were driving the results, given the number of times the visual cues were repeated after the disturbance, I would have expected the response to the visual cues to exceed the levels attained in the early stages of the trials. Additionally, the role of negative experiences in sensitization would predict higher overall levels of following behavior in those groups exposed to alarm cue. Figure 2b illustrates that neither of these conditions were met.

This study builds on previous work by indicating that prey fish use the first cue they receive, in this case chemical, to assign a context to subsequent ambiguous cues received by other modalities in order to respond to them in an appropriate, threat-sensitive manner. The observation that fish exposed to chemical cues indicative of predation also tend to respond more strongly to visual cues of predation (e.g., Wisenden et al. 2004) could therefore, at least in part, be due to an increase in responsiveness to visual cues in general. This work thus provides further evidence for the importance of multimodal cues in driving adaptive animal behavior (Rowe 1999; McLennan 2003).

## Ethical approval

All applicable international, national, and institutional guidelines for the care and use of animals were followed. All procedures performed involving animals were in accordance with the ethical standards of Cardiff University, the institution at which the study was conducted. This work was conducted under the UK Home Office license (PPL 30/2876) with approval by the Cardiff University Animal Ethics Committee. As described above, during the course of this experiment, fish were subjected to social isolation, abnormal lighting conditions, and startling stimuli. Although these factors are likely to have temporarily elevated their stress levels, no fish showed any signs of having suffered lasting harm and resumed normal behavior less than an hour after being returned to a breeding tank. Donor fish were not fed for 20 h during cue production. No lasting welfare effects of this treatment were observed or expected: guppies maintain normal behavior after 5 days of food deprivation (Archard et al. 2006). Throughout the maintenance and use of these fish, I adopted the principle that “the best animal welfare is a prerequisite for the best science,” following the “Guidelines for the treatment of animals in behavioral research and teaching” recommended by The Association for the Study of Animal Behaviour (2012).

## Electronic supplementary material

ESM 1 (DOCX 147 kb)
