# Supplementary material for: Keeping eyes peeled: guppies exposed to chemical alarm cue are more responsive to ambiguous visual cues
Source: Behav Ecol Sociobiol. 2016 Feb 23;70:575–84. doi: 10.1007/s00265-016-2076-4 (PMC4788695; doi:10.1007/s00265-016-2076-4)
Supplement: Supplementary file 1 — (DOCX 147 kb) [file 265_2016_2076_MOESM1_ESM.docx]

**Behavioral Ecology and Sociobiology Supplementary Material for:**

**Keeping eyes peeled: guppies exposed to chemical alarm cue are more responsive to ambiguous visual cues**

Jessica F. Stephenson^1,2,3^

^1^School of Biosciences, Cardiff University, Cardiff CF10 3AX, UK

^2^Center for Adaptation to a Changing Environment (ACE), ETH Zürich, Institute of Integrative Biology, 8092 Zürich, Switzerland

^3^EAWAG, Swiss Federal Institute of Aquatic Science and Technology, Department of Aquatic Ecology, 8600 Dübendorf, Switzerland

**Description**

In this supplementary material I provide further details on the analyses presented in the main text. For both models, I give the analysis of deviance table of the full starting model, each of the subsequent models during the stepwise deletion of non-significant terms, and the final model, the results of which I additionally report in the main text. At each step of the simplification process, the term I removed is highlighted in yellow, and I provide the likelihood ratio test comparing each model to the next, simplified one. In all cases, the AIC value drops during simplification, and no simplification removes a statistically important variable (as evidenced by the *P* values of over 0.05 in each likelihood ratio test).

For both models, I confirmed that the residuals conformed to the assumption of normality by visually inspecting histograms of the residuals from the full starting models and the final, simplified models. I provide these figures here (Figures S1-S4).

**MODEL 1: ACTIVITY – SQUARE ROOT TRANSFORMED**

**Full starting model:**

Analysis of Deviance Table (Type II Wald F tests with Kenward-Roger df)

Response: sqrt(activity)

F Df Df.res Pr(>F)

sex 0.6112 1 61.88 0.4373

time 98.4557 1 547.00 < 2.2e-16 ***

treatment 1.1639 3 60.43 0.3310

W 0.0014 1 50.83 0.9698

timestart 1.2432 1 64.74 0.2690

sex:time 0.0210 1 547.00 0.8848

time:treatment 10.5737 3 547.00 9.082e-07 ***

time:W 0.2013 1 547.00 0.6539

sex:W 0.0590 1 67.74 0.8088

sex:treatment 0.5251 3 62.80 0.6666

---

Signif. codes: 0 ‘***’ 0.001 ‘**’ 0.01 ‘*’ 0.05 ‘.’ 0.1 ‘ ’ 1

**Figure S1:** Histogram of residuals of the full, starting model of fish activity level.

**Remove sex:time interaction:**

Model2 is the simplified model:

Df AIC BIC logLik deviance Chisq Chi Df Pr(>Chisq)

model2 19 1739.6 1824.2 -850.82 1701.6

model1 20 1741.6 1830.6 -850.81 1701.6 0.0213 1 0.8841

Model2

Analysis of Deviance Table (Type II Wald F tests with Kenward-Roger df)

Response: sqrt(activity)

F Df Df.res Pr(>F)

sex 0.6112 1 61.88 0.4373

time 98.6319 1 548.00 < 2.2e-16 ***

treatment 1.1639 3 60.43 0.3310

W 0.0014 1 50.83 0.9698

timestart 1.2432 1 64.74 0.2690

time:treatment 10.5895 3 548.00 8.881e-07 ***

time:W 0.1982 1 548.00 0.6563

sex:W 0.0590 1 67.74 0.8088

sex:treatment 0.5251 3 62.80 0.6666

---

Signif. codes: 0 ‘***’ 0.001 ‘**’ 0.01 ‘*’ 0.05 ‘.’ 0.1 ‘ ’ 1

**Remove sex:weight interaction:**

Model3 is the simplified model:

Df AIC BIC logLik deviance Chisq Chi Df Pr(>Chisq)

model3 18 1737.7 1817.8 -850.85 1701.7

model2 19 1739.6 1824.2 -850.82 1701.6 0.0702 1 0.791

Model3

Analysis of Deviance Table (Type II Wald F tests with Kenward-Roger df)

Response: sqrt(activity)

F Df Df.res Pr(>F)

sex 0.6171 1 62.89 0.4351

time 98.6319 1 548.00 < 2.2e-16 ***

treatment 1.2613 3 61.43 0.2956

W 0.0014 1 51.51 0.9698

timestart 1.2077 1 64.91 0.2758

time:treatment 10.5895 3 548.00 8.881e-07 ***

time:W 0.1982 1 548.00 0.6563

sex:treatment 0.6112 3 64.20 0.6102

---

Signif. codes: 0 ‘***’ 0.001 ‘**’ 0.01 ‘*’ 0.05 ‘.’ 0.1 ‘ ’ 1

**Remove time:fish weight interaction:**

Model4 is the simplified model:

Df AIC BIC logLik deviance Chisq Chi Df Pr(>Chisq)

model4 17 1343.8 1417.4 -654.87 1309.8

model3 18 1344.7 1422.7 -654.36 1308.7 1.0273 1 0.3108

Model4

Analysis of Deviance Table (Type II Wald F tests with Kenward-Roger df)

Response: sqrt(activity)

F Df Df.res Pr(>F)

sex 0.6910 1 60.82 0.4091

time 120.8586 1 483.03 < 2.2e-16 ***

treatment 0.9178 3 58.30 0.4380

W 0.0311 1 33.87 0.8611

timestart 0.1283 1 63.13 0.7214

time:treatment 11.7664 3 483.04 1.883e-07 ***

sex:treatment 1.1912 3 61.90 0.3205

---

Signif. codes: 0 ‘***’ 0.001 ‘**’ 0.01 ‘*’ 0.05 ‘.’ 0.1 ‘ ’ 1

**Remove fish weight main effect:**

Model5 is the simplified model:

Df AIC BIC logLik deviance Chisq Chi Df Pr(>Chisq)

model5 16 1341.8 1411.1 -654.90 1309.8

model4 17 1343.8 1417.4 -654.87 1309.8 0.0433 1 0.8352

Model5

Analysis of Deviance Table (Type II Wald F tests with Kenward-Roger df)

Response: sqrt(activity)

F Df Df.res Pr(>F)

sex 0.7209 1 61.67 0.3991

time 120.8756 1 483.04 < 2.2e-16 ***

treatment 0.8968 3 58.11 0.4484

timestart 0.1547 1 64.36 0.6954

time:treatment 11.7676 3 483.06 1.88e-07 ***

sex:treatment 1.2596 3 62.09 0.2961

---

Signif. codes: 0 ‘***’ 0.001 ‘**’ 0.01 ‘*’ 0.05 ‘.’ 0.1 ‘ ’ 1

**Remove the the effect of the time at which the trial began:**

Model6 is the simplified model:

Df AIC BIC logLik deviance Chisq Chi Df Pr(>Chisq)

model6 15 1340.0 1404.9 -654.99 1310.0

model5 16 1341.8 1411.1 -654.90 1309.8 0.1855 1 0.6667

Model6

Analysis of Deviance Table (Type II Wald F tests with Kenward-Roger df)

Response: sqrt(activity)

F Df Df.res Pr(>F)

sex 0.9037 1 62.41 0.3454

time 120.8662 1 483.06 < 2.2e-16 ***

treatment 0.9744 3 58.90 0.4110

time:treatment 11.7689 3 483.08 1.877e-07 ***

sex:treatment 1.4133 3 63.02 0.2472

---

Signif. codes: 0 ‘***’ 0.001 ‘**’ 0.01 ‘*’ 0.05 ‘.’ 0.1 ‘ ’ 1

**Remove the sex:treatment interaction:**

Model7 is the simplified model:

Df AIC BIC logLik deviance Chisq Chi Df Pr(>Chisq)

model7 12 1338.7 1390.6 -657.33 1314.7

model6 15 1340.0 1404.9 -654.99 1310.0 4.6766 3 0.1971

Model7

Analysis of Deviance Table (Type II Wald F tests with Kenward-Roger df)

Response: sqrt(activity)

F Df Df.res Pr(>F)

sex 0.9109 1 65.69 0.3434

time 121.0739 1 483.11 < 2.2e-16 ***

treatment 0.9635 3 61.94 0.4157

time:treatment 11.7058 3 483.18 2.045e-07 ***

---

Signif. codes: 0 ‘***’ 0.001 ‘**’ 0.01 ‘*’ 0.05 ‘.’ 0.1 ‘ ’ 1

**Remove the sex main effect:**

Model8 is the simplified model:

Df AIC BIC logLik deviance Chisq Chi Df Pr(>Chisq)

model8 11 1337.7 1385.3 -657.82 1315.7

model7 12 1338.7 1390.6 -657.33 1314.7 0.9931 1 0.319

Model8

Analysis of Deviance Table (Type II Wald F tests with Kenward-Roger df)

Response: sqrt(activity)

F Df Df.res Pr(>F)

time 120.8841 1 483.20 < 2.2e-16 ***

treatment 0.9708 3 62.94 0.4122

time:treatment 11.7322 3 483.20 1.973e-07 ***

---

Signif. codes: 0 ‘***’ 0.001 ‘**’ 0.01 ‘*’ 0.05 ‘.’ 0.1 ‘ ’ 1

**Figure S2:** Histogram of residuals of the final, simplified model of fish activity level.

**MODEL 2: PROPORTION OF TIME SPENT FOLLOWING THE STRIPES – ARCSINE TRANSFORMED**

**Full starting model:**

Analysis of Deviance Table (Type II Wald F tests with Kenward-Roger df)

Response: asin(following)

F Df Df.res Pr(>F)

sex 1.6511 1 65.73 0.203320

time 8.3610 1 576.47 0.003978 **

activity 89.1123 1 546.45 < 2.2e-16 ***

treatment 0.8611 3 62.96 0.466035

W 3.0987 1 41.64 0.085699 .

timestart 0.1468 1 69.15 0.702817

sex:time 0.1640 1 575.19 0.685682

activity:treatment 4.4476 3 537.73 0.004239 **

time:activity 25.9300 1 560.77 4.842e-07 ***

sex:activity 1.6064 1 556.82 0.205531

time:W 1.4599 1 554.93 0.227455

sex:W 0.0690 1 67.77 0.793619

---

Signif. codes: 0 ‘***’ 0.001 ‘**’ 0.01 ‘*’ 0.05 ‘.’ 0.1 ‘ ’ 1

**Figure S3:** Histogram of residuals of the full, starting model of the proportion of time fish spent following the visual stimulus.

**Remove sex:weight interaction:**

Model2 is the simplified model:

Df AIC BIC logLik deviance Chisq Chi Df Pr(>Chisq)

model2 19 735.06 819.58 -348.53 697.06

model1 20 736.97 825.95 -348.49 696.97 0.0831 1 0.7731

Model2:

Analysis of Deviance Table (Type II Wald F tests with Kenward-Roger df)

Response: asin(following)

F Df Df.res Pr(>F)

sex 1.6757 1 66.69 0.199958

time 8.3355 1 576.83 0.004034 **

activity 89.7964 1 546.75 < 2.2e-16 ***

treatment 0.8099 3 63.95 0.493055

W 3.1548 1 42.81 0.082812 .

timestart 0.1883 1 69.18 0.665696

sex:time 0.1667 1 575.63 0.683180

activity:treatment 4.5690 3 536.51 0.003591 **

time:activity 25.9124 1 561.09 4.883e-07 ***

sex:activity 1.6129 1 556.43 0.204617

time:W 1.4695 1 555.10 0.225933

---

Signif. codes: 0 ‘***’ 0.001 ‘**’ 0.01 ‘*’ 0.05 ‘.’ 0.1 ‘ ’ 1

**Remove the effect of the time the experiment began:**

Model3 is the simplified model:

Df AIC BIC logLik deviance Chisq Chi Df Pr(>Chisq)

model3 18 733.27 813.35 -348.64 697.27

model2 19 735.06 819.58 -348.53 697.06 0.215 1 0.6429

Model3:

Analysis of Deviance Table (Type II Wald F tests with Kenward-Roger df)

Response: asin(following)

F Df Df.res Pr(>F)

sex 1.6287 1 67.62 0.206253

time 8.3833 1 577.36 0.003930 **

activity 90.3402 1 545.82 < 2.2e-16 ***

treatment 0.8751 3 64.61 0.458706

W 3.5495 1 43.17 0.066309 .

sex:time 0.1541 1 576.35 0.694755

activity:treatment 4.5372 3 536.00 0.003751 **

time:activity 25.8422 1 561.75 5.054e-07 ***

sex:activity 1.5283 1 554.67 0.216898

time:W 1.4515 1 555.29 0.228808

---

Signif. codes: 0 ‘***’ 0.001 ‘**’ 0.01 ‘*’ 0.05 ‘.’ 0.1 ‘ ’ 1

**Remove the sex:time interaction:**

Model4 is the simplified model:

Df AIC BIC logLik deviance Chisq Chi Df Pr(>Chisq)

model4 17 731.42 807.05 -348.71 697.42

model3 18 733.27 813.35 -348.64 697.27 0.1461 1 0.7023

Model4:

Analysis of Deviance Table (Type II Wald F tests with Kenward-Roger df)

Response: asin(following)

F Df Df.res Pr(>F)

sex 1.6305 1 67.67 0.205994

time 8.3917 1 578.42 0.003912 **

activity 90.6295 1 546.41 < 2.2e-16 ***

treatment 0.8787 3 64.62 0.456885

W 3.5513 1 43.20 0.066238 .

activity:treatment 4.5070 3 538.22 0.003908 **

time:activity 26.4360 1 561.70 3.768e-07 ***

sex:activity 1.3850 1 582.28 0.239735

time:W 1.4278 1 555.65 0.232626

---

Signif. codes: 0 ‘***’ 0.001 ‘**’ 0.01 ‘*’ 0.05 ‘.’ 0.1 ‘ ’ 1

**Remove the sex:activity interaction:**

Model5 is the simplified model:

Df AIC BIC logLik deviance Chisq Chi Df Pr(>Chisq)

model5 16 730.74 801.93 -349.37 698.74

model4 17 731.42 807.05 -348.71 697.42 1.3277 1 0.2492

Model5:

Analysis of Deviance Table (Type II Wald F tests with Kenward-Roger df)

Response: asin(following)

F Df Df.res Pr(>F)

sex 1.6490 1 68.01 0.203451

time 7.9783 1 580.62 0.004897 **

activity 90.9574 1 543.29 < 2.2e-16 ***

treatment 0.8883 3 64.61 0.452011

W 3.6347 1 43.32 0.063235 .

activity:treatment 4.2290 3 530.80 0.005720 **

time:activity 27.5903 1 562.34 2.13e-07 ***

time:W 1.1895 1 555.14 0.275897

---

Signif. codes: 0 ‘***’ 0.001 ‘**’ 0.01 ‘*’ 0.05 ‘.’ 0.1 ‘ ’ 1

**Remove the time:fish weight interaction:**

Model6 is the simplified model:

Df AIC BIC logLik deviance Chisq Chi Df Pr(>Chisq)

model6 15 729.95 796.69 -349.98 699.95

model5 16 730.74 801.93 -349.37 698.74 1.2103 1 0.2713

Model6

Analysis of Deviance Table (Type II Wald F tests with Kenward-Roger df)

Response: asin(following)

F Df Df.res Pr(>F)

sex 1.6619 1 68.02 0.201708

time 7.9744 1 581.65 0.004907 **

activity 91.0500 1 544.13 < 2.2e-16 ***

treatment 0.8875 3 64.62 0.452412

W 3.6213 1 43.32 0.063702 .

activity:treatment 3.9327 3 535.23 0.008561 **

time:activity 27.6009 1 563.35 2.118e-07 ***

---

Signif. codes: 0 ‘***’ 0.001 ‘**’ 0.01 ‘*’ 0.05 ‘.’ 0.1 ‘ ’ 1

**Figure S4:** Histogram of residuals of the final, simplified model of the proportion of time fish spent following the visual stimulus.
